# La miliaire cristalline généralisée: à propos d’un cas

**DOI:** 10.11604/pamj.2018.30.69.15383

**Published:** 2018-05-28

**Authors:** Ouiam El Anzi, Badreddine Hassam

**Affiliations:** 1 Service de Dermatologie et Vénérologie, Centre Hospitalier Universitaire Ibn Sina, Faculté de Médecine et de Pharmacie, Université Mohammed V, Rabat, Maroc

**Keywords:** Miliaire sudorale, miliaire cristalline, fièvre, Prickly heat, miliaria crystallina, fever

## Abstract

Prickly heat is a benign cutaneous manifestation due to the obstruction of the sweat ducts following excessive exposure to heat, humidity or hyperthermia. We report the case of a 70-year old diabetic female patient treated with insulin, hospitalized in the Intensive Care Unit due to acidocetosic coma secondary to pyelonephritis. The patient had diffuse clear vesicular lesions spread all over the body,(a) firm to palpation, on a healthy skin, suggesting miliaria crystallina. Miliaria crystallina is a benign cutaneous manifestation due to sweat retention secondary to obstruction of the sweat ducts. It is possible to distinguish between miliaria crystallina due to the obstruction in the stratum corneum, miliaria rubra due to the obstruction within the malpighian stratum and miliaria profunda due to an obstrusction in or below the dermoepidermal junction, depending on the level of obstruction. Miliaria crystallina heals spontaneously in a few hours giving rise to a desquamation, as occurred in our patient after regression of febrile syndrome.

## Image en médecine

**Figure 1:**
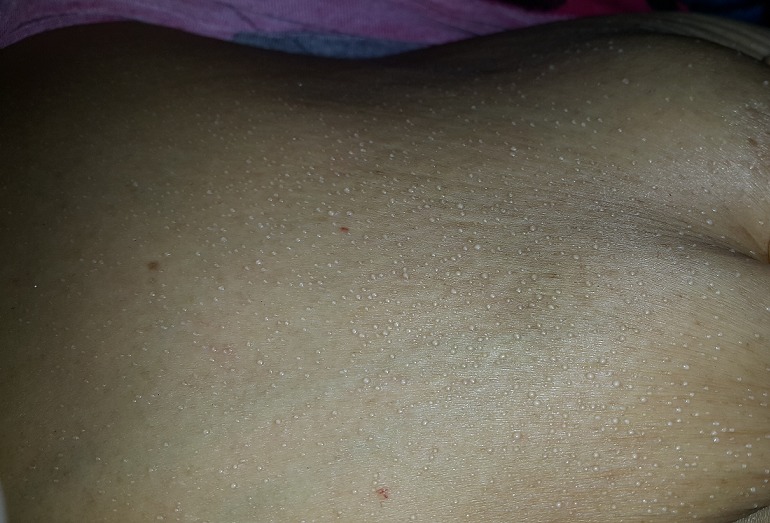
vésicules claires reposant sur une peau saine

La miliaire sudorale est une manifestation cutanée bénigne liée à l’obstruction sudorale généralement témoin d’une exposition excessive à la chaleur, à l’humidité ambiante ou d’une hyperthermie. Nous rapportons le cas d’une patiente de 70 ans, diabétique sous insuline, hospitalisée au service de réanimation pour un coma acidocétosique secondaire à une pyélonéphrite. La patiente a présenté des lésions vésiculeuses diffuses au niveau de tout le corps de contenu clair, fermes à la palpation, reposant sur une peau saine, correspondant à une miliaire cristalline. La miliaire sudorale est une manifestation cutanée bénigne liée à une rétention sudorale secondaire à une obstruction des canaux sudoraux. Selon le niveau d'obstruction, on distingue la miliaire cristalline par obstruction dans le stratum cornéum; la miliaire rouge par obstruction dans le stratum malpighi et la miliaire profonde par obstruction au niveau ou en dessous de la jonction dermo-épidermique. La miliaire cristalline guérit spontanément en quelques heures en laissant une desquamation, comme c'était le cas chez notre patiente après régression du syndrome fébrile. Figure 1

